# Transcriptome and Metabolome Analyses Reveal the Molecular Relationship Between Dietary Crude Protein Level and Liver Metabolism in Fattening Hu Sheep

**DOI:** 10.3390/metabo16060375

**Published:** 2026-05-29

**Authors:** Patiguli Abudukeyimu, Fengmei Xie, Yifan Hu, Haiying He, Cheng Hou, Yiming Sulaiman, Huiguo Yang, Gao Gong

**Affiliations:** 1 College of Animal Science, Xinjiang Agricultural University, Urumqi 830052, China; 19229012647@163.com (P.A.); 15083313151@163.com (Y.H.); 15299681902@163.com (C.H.); ysulaiman@xjau.edu.cn (Y.S.); 2 BiBang Sheep Industry Development Co., Ltd., Hotan 848102, China; 15809038782@163.com; 3 Xinjiang Academy of Animal Husbandry Sciences, Urumqi 830011, China; 15733286826@163.com; 4 Key Laboratory of Xinjiang Sheep Breeding Resources Innovation for Multiple Lambs, Hotan 848102, China

**Keywords:** fattening Hu sheep, crude protein, liver, transcriptomics, metabolomics, combined multi-omics analysis

## Abstract

**Highlights:**

- Q: What dietary crude protein level supports optimal growth of 4-month-old fattening Hu sheep under isoenergetic conditions in southern Xinjiang?
- A: 15.11% dietary crude protein (with ME = 9.4 MJ/kg DM) supports favorable growth performance.
- Q: What key hepatic regulatory pathways are identified via combined transcriptome-metabolome analysis?
- A: AMPK signaling, steroid biosynthesis, and oxidative phosphorylation are the core regulatory pathways.
- Q: Which hub genes and metabolites are tightly associated with average daily gain in Hu sheep?
- A: Hub genes ATP6AP1/LOC101119853 and metabolites calcidiol/ADP are strongly correlated with ADG.
- Q: How does graded dietary crude protein affect hepatic metabolism and stress resilience in Hu sheep?
- A: It induces hepatic metabolic reprogramming to maintain energy homeostasis and enhance arid environmental stress resilience.

**Abstract:**

**Background:** Dietary crude protein (CP) acts as a key nutritional factor that affects the growth performance and liver metabolism of fattening Hu sheep, with metabolizable energy (ME) representing a major confounding factor in CP-related responses. To isolate the specific effects of CP on liver metabolism and minimize energy–protein interactions, we standardized dietary ME at 9.4 MJ/kg dry matter. **Methods:** We then established three isoenergetic CP concentrations: 11.07%, 13.07%, and 15.11%. A total of ninety 4-month-old male Hu sheep (with an initial body weight of 27.09 ± 1.83 kg) were allocated at random to three dietary treatment groups, each containing 30 animals distributed across three replicate pens, and fed pelleted total mixed rations (PTMRs) for 75 days under pen conditions in southern Xinjiang. Exploratory combined transcriptomic and metabolomic profiling of liver tissue was conducted to characterize how graded CP levels modulate growth traits and hepatic metabolic pathways, thereby identifying the appropriate dietary CP level for efficient and sustainable fattening of Hu sheep in this region. **Result:** Results indicated that animals fed the 15.11% CP diet showed a significantly higher average daily gain (ADG) and cumulative weight gain compared with those fed 11.07% or 13.07% CP (*p* < 0.05). Exploratory multi-omics enrichment analysis demonstrated significant overrepresentation (*p* < 0.05) of differentially expressed genes and metabolites in key biological pathways—including bile secretion, AMP-activated protein kinase (AMPK) signaling, steroid biosynthesis, peroxisome proliferator-activated receptor (PPAR) signaling, and oxidative stress-related and oxidative phosphorylation. Correlation analyses characterized two hub genes—ATP6AP1 and LOC101119853—that were significantly and negatively correlated with ADG (*p* < 0.05), whereas two metabolites—calcidiol and ADP—displayed significant positive relationships with ADG (*p* < 0.05). Pathway-level comparisons further demonstrated that both the 13.07% vs. 15.11% CP and the 11.07% vs. 15.11% CP contrasts yielded significant enrichment in AMPK signaling and steroid biosynthesis. Notably, calcidiol and ADP both declined numerically in the 13.07% vs. 15.11% CP comparison, whereas only ADP reached statistical significance in the 11.07% vs. 15.11% CP contrast. **Conclusions:** Collectively, under an ME level of 9.4 MJ/kg, a dietary CP concentration of 15.11% contributes to favorable growth of 4-month-old fattening Hu sheep housed in pens in southern Xinjiang. This level is associated with improved growth performance and coordinated regulation of central hepatic regulatory networks—particularly those involved in energy homeostasis and steroidogenesis—thereby supporting metabolic stability without compromising animal health or production efficiency. These findings provide a preliminary molecular basis for precision protein nutrition in Hu sheep feeding systems and offer translational insights for optimizing ruminant nutrition under arid and semi-arid environmental constraints. All correlations indicate potential associations, not causal relationships.

## 1. Introduction

The Hu sheep (*Ovis aries*) is a Chinese indigenous breed known for its high fecundity, rapid growth, and strong adaptability to low-quality forages. It serves as a cornerstone breed for intensive meat sheep production and plays a pivotal role in advancing the meat sheep industry and rural revitalization initiatives in southern Xinjiang [1]. Dietary crude protein (CP) concentration represents a primary nutritional determinant affecting growth performance, nitrogen utilization efficiency, and metabolic health in fattening ruminants. Suboptimal CP intake compromises skeletal muscle accretion, impairs immune competence, and delays developmental milestones; conversely, excessive CP supplementation elevates feed formulation costs, imposes metabolic stress on the liver and kidneys, and promotes systemic oxidative damage [2,3,4]. Therefore, identifying the physiologically appropriate CP level—capable of maximizing nitrogen retention, stimulating myofibrillar protein synthesis, and sustaining whole-body metabolic homeostasis—is fundamental to precision feeding strategies for meat sheep [5,6].

Southern Xinjiang exhibits an arid continental climate characterized by extreme diurnal temperature fluctuations, low humidity, and recurrent sandstorms. These environmental stressors markedly influence nutrient requirements, resulting in distinct physiological and metabolic demands for Hu sheep relative to populations reared in more temperate inland regions. Despite its regional importance, evidence-based recommendations for dietary CP levels tailored to fattening Hu sheep under local pen-feeding conditions remain scarce. To date, most investigations have focused on phenotypic endpoints—including body weight gain, feed efficiency, and serum biochemical indices [7]—while the molecular underpinnings of CP-mediated regulation of hepatic metabolism remain uncharacterized.

The liver constitutes the central hub for intermediary metabolism and innate immune modulation in ruminants, orchestrating critical processes such as amino acid catabolism, ureagenesis, gluconeogenesis, and lipid flux regulation. As the principal sensor of dietary amino acid availability, it initiates adaptive transcriptional and metabolic changes in response to protein nutrition status; consequently, hepatic functional integrity directly governs dietary CP utilization efficiency and ultimately dictates animal growth outcomes [8,9]. Accumulating evidence demonstrates that dietary CP levels modulate hepatic redox balance, immunometabolic signaling, and metabolic flexibility through coordinated alterations in metabolite pools and expression of regulatory genes involved in energy sensing and biosynthetic pathways [10]. Notably, AMP-activated protein kinase (AMPK) functions as a master cellular energy rheostat, integrating nutrient and hormonal signals to maintain hepatic energetic homeostasis—making it a justified focal point for this investigation [11].

Multi-omics technologies have shifted ruminant nutritional research from single-pathway analyses to integrative, systems-level understanding. Transcriptomics enables comprehensive profiling of transcriptional dynamics across the genome, whereas metabolomics provides quantitative resolution of endogenous small-molecule metabolites reflecting real-time physiological states. Their synergistic integration establishes an associative framework linking gene expression changes→metabolite perturbations→phenotypic outcomes—a powerful strategy for deciphering nutrient–host interactions at molecular resolution [12,13,14]. To date, however, no study has systematically delineated the transcriptome–metabolome crosstalk underlying hepatic metabolic adaptation to graded CP levels in fattening Hu sheep from southern Xinjiang, under rigorously controlled isoenergetic conditions (ME fixed at 9.4 MJ/kg DM) designed to eliminate confounding energy–protein interactions.

To fill this knowledge gap, the present study employed a controlled feeding trial using 4-month-old intact male Hu sheep (*n* = 90), assigned to three isoenergetic diets containing 11.07%, 13.07%, or 15.11% CP (dry matter basis), all formulated as pelleted total mixed rations (PTMRs). Leveraging paired liver tissue transcriptomic and metabolomic profiling, we aimed to: (i) quantify current study used CP content on growth traits under standardized energy supply; (ii) characterize the hierarchical molecular responses—spanning transcriptional regulation and metabolic flux—that mediate hepatic metabolic homeostasis; and (iii) determine the appropriate CP concentration supporting favorable growth efficiency without compromising metabolic health, thereby informing evidence-based, precision nutrition guidelines for Hu sheep production in arid agroecosystems.

## 2. Materials and Methods

### 2.1. Ethical Statement

All animal-related experimental procedures involving animals were performed in full accordance with the Guide for the Care and Use of Laboratory Animals issued by the Ministry of Science and Technology of China (2006), and were formally approved by the Institutional Animal Care and Use Committee (IACUC) at the Institute of Animal Science, Xinjiang Academy of Animal Husbandry Sciences (Approval No.: 202505-01). Housing, husbandry, and environmental conditions complied with the national standard Laboratory Animal—Environment and Housing Facilities (GB 14925-2010) [15], and reporting adhered to the ARRIVE 2.0 framework to ensure methodological transparency and ethical rigor.

### 2.2. Experimental Animals, Feeding Management, and Diets

The animal feeding trial was carried out at the BiBang Sheep Industry Development Co., Ltd. experimental base in Hotan, Xinjiang. A total of ninety 4-month-old intact male Hu sheep were randomly allocated to three dietary treatment groups (*n* = 3 pens per group, 10 sheep per pen), with three replicate pens per treatment. The treatments consisted of pelleted total mixed rations (PTMR) formulated to contain 11.07% CP (Group I), 13.07% CP (Group II), or 15.11% CP (Group III) on a dry matter basis, while metabolizable energy (ME) was strictly standardized at 9.4 MJ/kg DM across all diets. Diet formulations adhered to the Chinese national standard Nutrient Requirements of Sheep (NY/T 816–2021) [15] and the U.S. National Research Council’s Nutrient Requirements of Ruminants (NRC, 2007) [16]. The ingredient composition and measured nutrient contents of the experimental diets are listed in Table 1.

Animals were housed in fully enclosed, climate-adapted, standardized sheep pens equipped with individual feed bunks and automatic waterers. The total experimental duration spanned 75 days, consisting of a 15-day acclimation period—including anthelmintic administration, routine vaccination, and stepwise diet transition—and a subsequent 60-day principal feeding period. Sheep were offered feed twice daily at 09:00 and 19:00; both feed and fresh water were available ad libitum. Growth performance data (including initial/final body weight, average daily gain, and feed efficiency ratio) were collected concurrently during the same feeding trial and have been previously reported in a peer-reviewed publication [17]; formal permission for secondary use of these phenotypic datasets was obtained from the original journal publisher. The present study exclusively utilized liver tissue samples from this cohort to investigate the molecular relationships underlying CP-related regulation of hepatic metabolism.

### 2.3. Determination of Dietary Nutrient Composition

Dietary dry matter (DM) content was measured gravimetrically by oven-drying at 105 °C until a constant weight was achieved, in accordance with GB/T 6435–2006 [18]. Crude protein (CP) concentration was quantified via Kjeldahl nitrogen analysis (N × 6.25), per GB/T 6432–2018 [19]. Neutral detergent fiber (NDF) and acid detergent fiber (ADF) levels were determined using an ANKOM Fiber Analyzer (ANKOM Technology, Macedon, NY, USA) with heat-stable α-amylase and sodium sulfite, following the procedures described in GB/T 20806–2022 [18] and NY/T 1452–2022 [20], respectively. Calcium (Ca) and phosphorus (P) concentrations were quantified via inductively coupled plasma optical emission spectrometry (ICP-OES) after nitric–perchloric acid digestion, as specified in GB/T 6436–2018 [21] and GB/T 6437–2018 [22]. Metabolizable energy (ME) values were estimated using the NRC (2007) [16] prediction equations for ruminants, based on ingredient-specific ME coefficients sourced from the Chinese Feed Composition and Nutritional Value Table (31st Edition, 2020). The calculation was performed using the weighted summation formula:

ME (MJ/kg) = Σ (A × B)

In the formula: A represents the metabolizable energy of a certain feed ingredient (MJ/kg, referring to “China Feed Composition and Nutritional Value Table (31st Edition, 2020)”); B represents the percentage of this feed ingredient in the diet (%).

### 2.4. Tissue Sampling Procedure

On day 60 of the principal feeding period, six clinically healthy sheep per treatment group—selected to approximate the group mean body weight within ±5%—were subjected to a 12 h post absorptive fast (with unrestricted access to clean drinking water) prior to humane euthanasia. Liver tissue was harvested immediately post mortem: approximately 2.0 ± 0.2 g of the right lateral lobe was excised under sterile conditions, rinsed thoroughly with ice-cold 0.9% physiological saline to eliminate residual blood, rapidly minced into homogeneous fragments on a pre-chilled stainless-steel surface, and aliquoted into RNase/DNase-free cryovials. Samples were flash-frozen in liquid nitrogen for ≥10 min and subsequently stored at −80 °C until transcriptomic and metabolomic analyses. All procedures were conducted on ice or at 4 °C to minimize RNA degradation and metabolite turnover.

### 2.5. Transcriptomic Profiling

The liver tissue was subjected to the total RNA isolation using TRIzol^®^ Reagent (Invitrogen, Carlsbad, CA, USA) as per its instructions. The concentration and purity of RNA were determined spectrophotometrically using a NanoDrop 2000 UV-Vis Spectrophotometer (Thermo Fisher Scientific, Wilmington, DE, USA) with an A260/A280 ratio of 1.8230/2.0 being considered pure and chosen to proceed with the experiment. The integrity of RNAs was carefully assessed through the capillary electrophoresis on an Agilent 2100 Bioanalyzer (Agilent Technologies, Santa Clara, CA, USA); an Illumina NovaSeq 6000 platform (Illumina Inc., San Diego, CA, USA) was used to perform strand-specific cDNA libraries with 1 μg of high-quality total RNA using the Illumina TruSeq Stranded mRNA LT Sample Prep Kit (Illumina Inc., San Diego, CA, USA); 150 bp paired-end sequencing of the library was carried out.

Raw reads of the sequencing were quality controlled using FastQC v0.11. 9; Trimmomatic v0.39 was used to remove adapter sequences and low-quality bases (Phred score < 20). HISAT2 v2.2 was used to map clean reads to the most recent ovine reference genome (Oar_v4.0 GCA 017524585.1) [23,24]. Gene-level expression quantification was performed with StringTie v2.2.1, reporting transcripts per million (TPM) and fragments per kilobase of transcript per million mapped reads (FPKM) values. Differentially expressed genes (DEGs) were identified using DESeq2 v1.38.3 [25], with statistical significance defined as |log2(fold change)| ≥ 0.585 (equivalent to ≥1.5-fold change) and false discovery rate (FDR) < 0.05. Functional enrichment analyses, including Gene Ontology (GO) annotation and Kyoto Encyclopedia of Genes and Genomes (KEGG) pathway enrichment, were performed using R software (version 4.3.1) using the clusterProfiler package. De novo transcript assembly and annotation of novel isoforms were performed using StringTie in conjunction with Cuffcompare against the reference annotation (Ensembl Oar_v4.0).

### 2.6. Untargeted Hepatic Metabolomic Profiling

Liver metabolites were profiled in this work by liquid chromatography–mass spectrometry (LC-MS). Waters Acquity I-Class PLUS UPLC-Xevo G2-XS QTOF (Waters, Milford, MA, USA) ultra-high performance liquid chromatography–tandem mass spectrometry system was used to perform the metabolomics analysis. The separation in chromatography was accomplished with the help of an Acquity UPLC HSS T3 column (1.8 μm, 2.1 × 100 mm; Waters). A system was equipped with a source of electrospray ionization that was heated (Thermo Fisher Scientific, Waltham, MA, USA). Sample analysis was done in both positive and negative ionization modes (column: C18 column, 2.1 mm × 100 mm, 1.9 mm; mobile phase: A consisted of 0.1% formic acid in water, and mobile phase B consisted of 0.1% formic acid in acetone; gradient elution program was set as follows: 0–10 min 5–95 percent B; flow rate, 0.3 mL/min; column temperature, 40 °C; injection volume, 2 mL). The freeze-dried liver samples were thawed in an ice bath (after 12 h) and 50 mg of freeze-dried powder was added to a 1 mL of 80% methanol–water solution (with 0.1% formic acid) and ultrasonically extracted at room temperature in an ice bath (15 min), frozen at −20 °C (30 min) and centrifuged at 4 °C, 12,000 rpm (15 min). An organic filter (0.22 μm) was used to filter the supernatant and keep it in −80 °C. L-2-chlorophenylalanine (0.06 mg/mL, dissolved in methanol) was added as an internal standard. All samples were mixed in equal volumes to create quality control (QC) samples and one of the QC samples was added to every 10 samples [26]. Progenesis QI v2.3 (Nonlinear Dynamics, Newcastle, UK) was used to process the raw data. Metabolites were putatively identified by matching to KEGG, HMDB (v5.0), and LIPID MAPS (v2.0) databases. According to the Metabolomics Standards Initiative (MSI), the confidence level is 2 (putative identification); no authentic standards were used. Quantification was based on peak area. Differentially expressed metabolites (DEMs) were defined as |FC| ≥ 1.5, VIP > 1, and *p* < 0.05. Principal component analysis (PCA) was used to assess model stability [27]. OPLS-DA was performed using the R package ropls; model quality was evaluated by R^2^Y(cum) and Q^2^(cum) from 7-fold cross-validation. Overfitting was tested by 200 permutation tests, and a model was considered valid if the Q^2^ intercept was <0.

### 2.7. Joint Analysis

The transcriptome and metabolome data were subjected to KEGG co-enrichment [28]. The differentially expressed genes within the pathways and all the differentially expressed metabolites were selected. The Spearman correlation coefficient between DEGs and DEMs was calculated using the cor function in R language. Gene–metabolite pairs with a correlation coefficient >0.8 were screened out to construct a correlation-based association network. The joint biological annotation was conducted in combination with the KEGG database.

### 2.8. Statistical Analysis

For the growth performance data, each experiment was conducted with a single pen as the experimental unit (*n* = 3 pens per group), and one-way analysis of variance (ANOVA) and the least significant difference method (LSD) were used for post hoc multiple comparisons. For the transcriptome and metabolome data of liver tissue, since this was an exploratory mechanistic study aimed at capturing the treatment effect to the greatest extent, we temporarily used individual animals (*n* = 6 per group) as the analysis unit for the screening of differentially expressed genes and metabolites. This approach may not have completely eliminated the random variation among individual animals within each pen, which is a limitation of this exploratory study (see Section 4). The threshold for differential screening was uniformly set as follows: for transcriptome data, |log_2_(multiple change)| ≥ 0.585 (1.5-fold) and false discovery rate (FDR) < 0.05; for metabolome data, variable projection importance (VIP) > 1.0, |log_2_(multiple change)| ≥ 0.585, and *p* < 0.05.

Prior to the experiment, the sample size was determined based on the typical population variation and practical feeding conditions in pen-housed sheep studies. In this exploratory study, we did not conduct a formal statistical power calculation; instead, statistical reliability was ensured by adopting pen as the experimental unit (*n* = 3 pens per group), with sufficient replication and consistent management to minimize environmental variation. All data were analyzed using appropriate statistical models to account for pen-level effects, ensuring the robustness of phenotypic comparisons.

## 3. Results

### 3.1. Effects of Dietary Crude Protein Level on Growth Performance in Fattening Hu Sheep

Group III (15.11% CP) exhibited significantly higher ADG than Groups I and II (*p* < 0.05). Although Group III showed numerically higher cumulative weight gain than Group II, the difference was not statistically significant (*p* = 0.072), indicating a plateau effect at the highest CP level [17].

### 3.2. Quality Assessment of Multi-Omics Datasets

**Figure 1:**
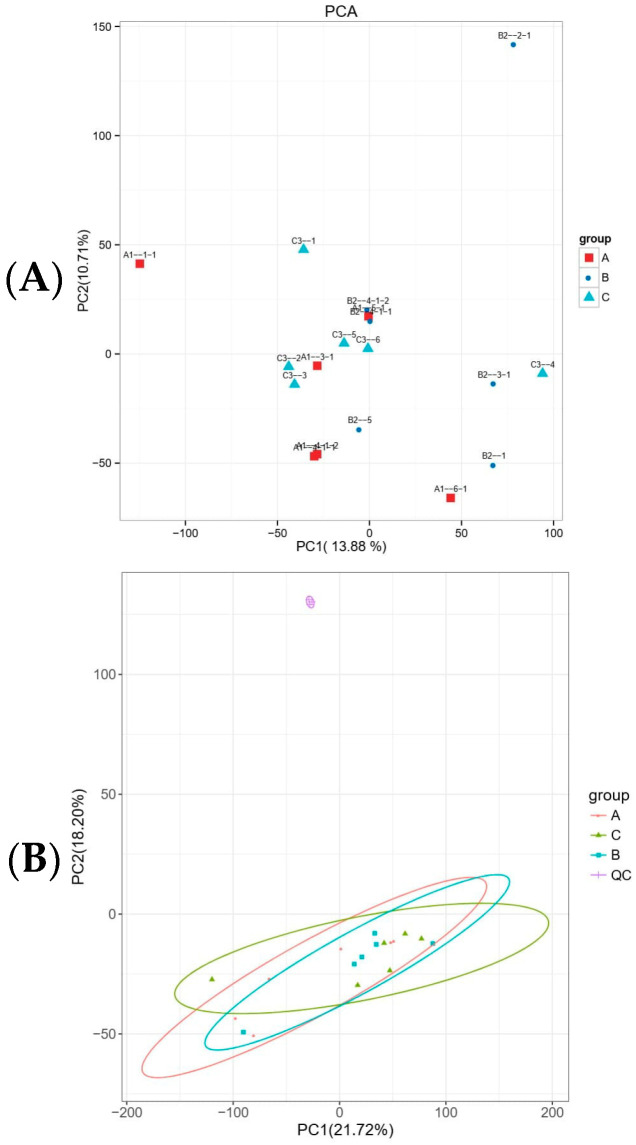
Overall overview of transcriptomic and metabolomic data. (**A**,**B**) Principal component analysis (PCA) plots of transcriptome and metabolome. Note: Groups A, B, and C represent the 11.07% CP (Group I), 13.07% CP (Group II), and 15.11% CP (Group III) treatment groups, respectively. The *x*-axis denotes principal component 1 (PC1), explaining 21.72% of total variance; the *y*-axis denotes principal component 2 (PC2), explaining 18.20% of total variance. Quality control (QC) samples—prepared by pooling equal volumes of all experimental extracts—are clustered centrally, confirming analytical stability and batch consistency.

Transcriptomic sequencing generated 117.72 Gb of high-fidelity clean reads across all samples. Per-sample Q30 scores exceeded 97.83%, and GC content ranged narrowly between 48.52% and 50.15%, indicating minimal sequencing bias and excellent base-call accuracy. Alignment rates to the ovine reference genome (*Ovis aries* Oar_v4.0, GCA_017524585.1) ranged from 96.69% to 98.26%—all well above the 90% threshold recommended for robust differential expression analysis. Principal component analysis (PCA) was conducted on the transcriptomic expression profiles of samples from groups A, B, and C to evaluate the overall inter-group differences and intra-group repeatability. The first principal component (PC1) explained 13.88% of the total variance among samples, while the second principal component (PC2) explained 10.71% of the variance. The cumulative contribution rate of the two components was 24.59%, which could reflect the main variation characteristics of the transcriptomic profiles (Figure 1A).

In untargeted metabolomics, OPLS-DA score plots showed clear separation among the three CP groups. For Group I and III, the model had R^2^Y(cum) = 0.992 and Q^2^(cum) = 0.857. Permutation tests (200 times) yielded a negative Q^2^ intercept (−0.261), and all permuted Q^2^ values were lower than the original Q^2^, confirming no overfitting and good predictive ability (Figure S1). In addition, quality control (QC) samples clustered tightly in the center of the principal component analysis (PCA) score plot (Figure 1B), demonstrating high analytical reproducibility and system stability throughout the batch run. The first two principal components—PC1 (21.72%) and PC2 (18.20%)—together accounted for 39.92% of total variance. A clear gradient separation was observed along PC1: Group I (11.07% CP) and Group III (15.11% CP) occupied opposing ends of the axis, while Group II (13.07% CP) localized intermediately, reflecting gradual remodeling of the hepatic metabolome in response to graded dietary CP levels.

### 3.3. Differential Gene Expression Analysis

**Figure 2:**
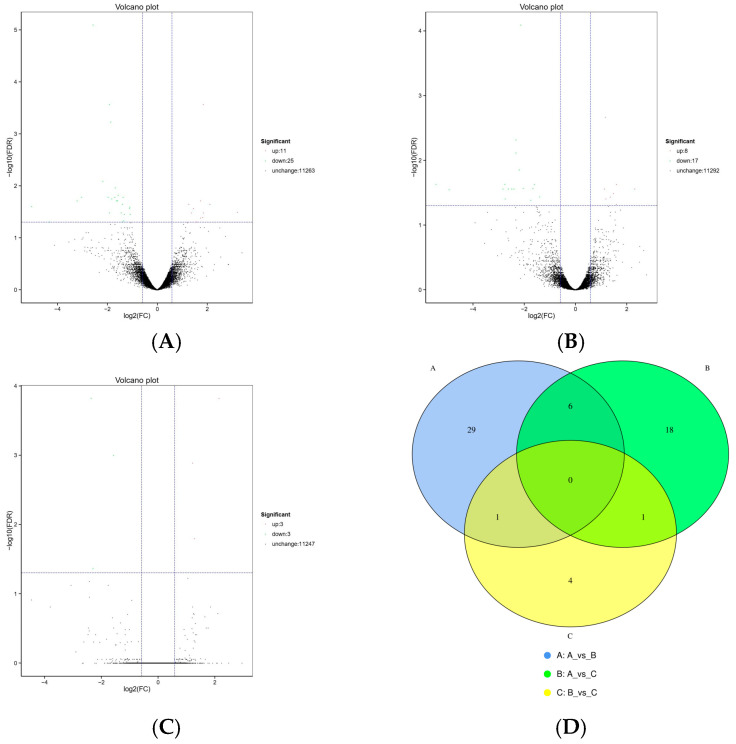
Differential gene expression profiling in liver tissue across dietary crude protein treatments. Note: Panels (**A**–**C**) present volcano plots depicting log2(fold change) versus –log10(FDR) for differentially expressed genes (DEGs) characterized in pairwise comparisons: (**A**), Group I (11.07% CP) vs. Group II (13.07% CP); (**B**), Group I vs. Group III (15.11% CP); (**C**), Group II vs. Group III. Red and blue points denote up-regulated and down-regulated DEGs, respectively (|log2(fold change)| ≥ 0.585, FDR < 0.05). Panel (**D**) shows the Venn diagram illustrating the overlap of unique DEGs across all three pairwise contrasts, with the central intersection representing genes differentially expressed in all comparisons.

Differentially expressed genes (DEGs) were characterized using DESeq2 v1.38.3 with stringent statistical thresholds: |log2(fold change)| ≥ 0.585 (≥1.5-fold) and false discovery rate (FDR) < 0.05. Pairwise comparisons demonstrated the following numbers of DEGs: 36 between Group I (11.07% CP) and Group II (13.07% CP), comprising 11 up-regulated and 25 down-regulated genes; 25 between Group I and Group III (15.11% CP), comprising 8 up-regulated and 17 down-regulated genes; and only 6 between Group II and Group III, comprising 3 up-regulated and 3 down-regulated genes. The union set across all three pairwise contrasts comprised 67 unique DEGs (Figure 2), indicating that the largest transcriptional divergence occurred at the extremes of the CP gradient—particularly between the lowest and highest dietary protein levels.

### 3.4. Functional Enrichment Analysis of Differentially Expressed Genes

Gene Ontology (GO) enrichment analysis demonstrated distinct biological themes across pairwise comparisons. DEGs between Group I (11.07% CP) and Group II (13.07% CP) were significantly enriched (FDR < 0.05) in processes related to amino acid metabolism, lipid metabolism, and cellular energy homeostasis. In contrast, DEGs between Group I and Group III (15.11% CP) showed broader and more pronounced enrichment—spanning modified amino acid metabolism, organic nitrogen compound metabolic processes (including amino group transfer and nitrogen assimilation), mitochondrial respiratory chain function, oxidative stress response, and glutathione-mediated redox homeostasis. DEGs between Group II and Group III were predominantly associated with amino acid catabolism, lipid binding and intracellular transport, and regulation of oxidative stress defense mechanisms (all FDR < 0.05).

**Figure 3:**
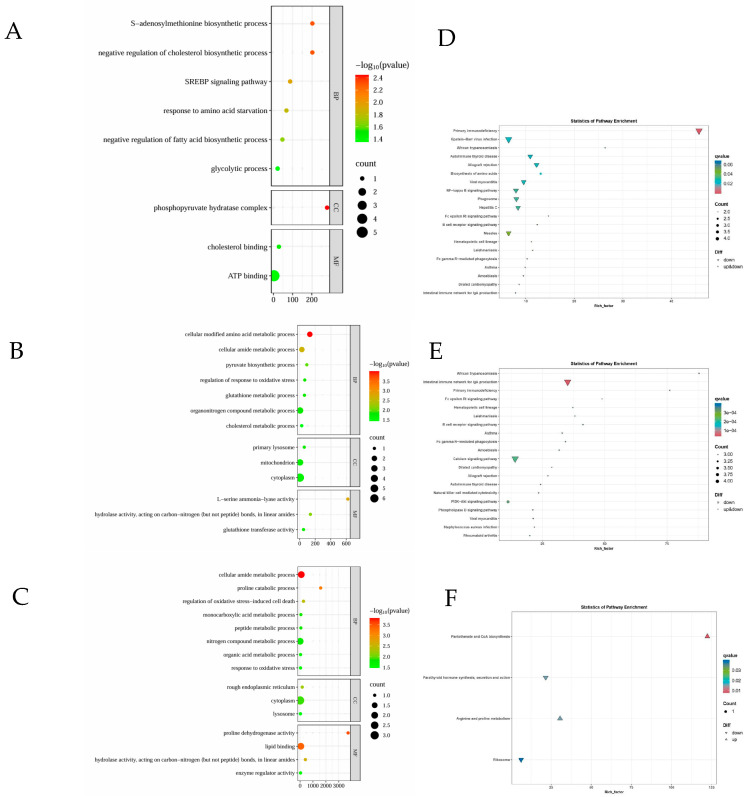
Functional enrichment analysis of differentially expressed genes (DEGs) in liver tissue: GO biological process terms and KEGG pathways. Note: Panels (**A**–**C**) display bubble plots for Gene Ontology (GO) biological process enrichment (Group I vs. Group II; Group I vs. Group III; Group II vs. Group III, respectively). Panels (**D**–**F**) present corresponding KEGG pathway enrichment results for the same comparisons. The *x*-axis represents the enrichment factor (ratio of observed to expected DEG count); the *y*-axis lists significantly enriched terms/pathways (FDR < 0.05). Bubble size reflects the number of DEGs mapped to each term/pathway; color intensity (blue to red) indicates the significance level of enrichment, with darker red representing lower FDR values.

KEGG pathway mapping corroborated and extended these findings. The most significantly enriched pathway for Group I vs. Group II DEGs was “Biosynthesis of amino acids” (ko01230), alongside immune-modulatory pathways including “NF-κB signaling” (ko04668). For Group I vs. Group III, enrichment spanned 12 highly significant pathways (FDR < 0.01), notably “PI3K-Akt signaling” (ko04151), “NF-κB signaling” (ko04668), “Branched-chain amino acid biosynthesis” (ko00290), “Pantothenate and CoA biosynthesis” (ko00650), “Glycine, serine and threonine metabolism” (ko00260), “Cysteine and methionine metabolism” (ko00270), “Glutathione metabolism” (ko00480), “Carbon metabolism” (ko01200), “Insulin signaling” (ko04910), and “mTOR signaling” (ko04150). DEGs between Group II and Group III were significantly enriched only in “Pantothenate and CoA biosynthesis” (ko00650) and “Arginine and proline metabolism” (ko00330) (FDR < 0.05; Figure 3). Collectively, these results indicate a gradual intensification of transcriptional reprogramming—particularly in amino acid handling, redox regulation, and growth-signaling cascades—as dietary CP increased from 11.07% to 15.11%.

### 3.5. Untargeted Hepatic Metabolomic Profiling and Differential Metabolite Identification

**Figure 4:**
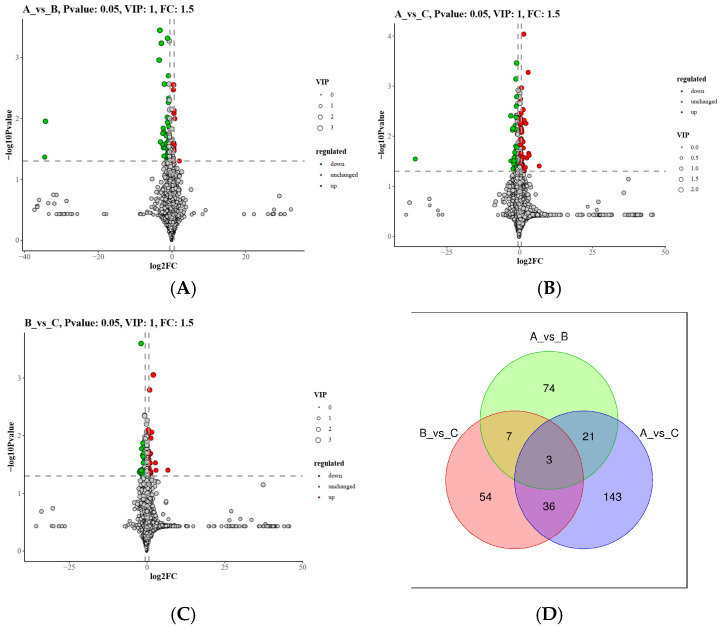
Differential metabolite profiling in liver tissue across dietary crude protein treatments. Note: Panels (**A**–**C**) display volcano plots of log2(fold change) versus −log10(FDR) for differentially expressed metabolites (DEMs) characterized in pairwise comparisons: (**A**), Group I (11.07% CP) vs. Group II (13.07% CP); (**B**), Group I vs. Group III (15.11% CP); (**C**), Group II vs. Group III. Red and green points denote metabolites significantly up-regulated and down-regulated, respectively (|log2(fold change)| ≥ 0.585, FDR < 0.05); black points represent non-significant features meeting neither criterion; (**D**) Venn diagram of Differential metabolites sets. The Venn diagram shows the overlap of differential metabolites identified from three pairwise comparisons: A vs. B (green), A vs. C (blue), and B vs. C (red).

Liquid chromatography–tandem mass spectrometry (LC–MS/MS) detected a total of 17,201 high-confidence metabolic features across all liver samples; of these, 3886 metabolites were confidently annotated using orthogonal database matching (KEGG, HMDB v5.0, LIPID MAPS v2.0) and MS/MS spectral validation. Differentially expressed metabolites (DEMs) were rigorously defined using three complementary criteria: |log2(fold change)| ≥ 0.585 (≥1.5-fold), variable importance in projection (VIP) > 1.0 from OPLS-DA modeling, and *p* < 0.05 (two-tailed Student’s *t*-test, FDR-corrected). Pairwise comparisons yielded the following numbers of DEMs: 51 between Group I (11.07% CP) and Group II (13.07% CP), comprising 15 up-regulated and 36 down-regulated metabolites; 108 between Group I and Group III (15.11% CP), comprising 60 up-regulated and 48 down-regulated metabolites; and 49 between Group II and Group III, comprising 25 up-regulated and 24 down-regulated metabolites. The union set across all three contrasts comprised 208 unique DEMs (Figure 4), with the largest metabolic perturbation observed between the lowest and highest dietary crude protein levels.

### 3.6. KEGG Pathway Enrichment Analysis of Differentially Expressed Metabolites

**Figure 5:**
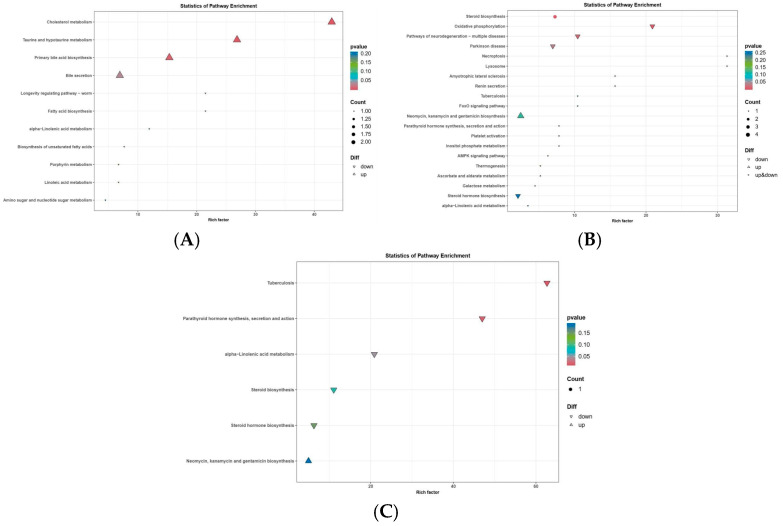
(**A**) KEGG pathway enrichment analysis of differentially expressed metabolites (DEMs) between Group I and Group III. (**B**) KEGG pathway enrichment analysis of differentially expressed metabolites (DEMs) between Group II and Group III. (**C**) KEGG pathway enrichment analysis of differentially expressed metabolites (DEMs) between Group II and Group III. The *x*-axis represents the enrichment factor (Rich factor), calculated as the ratio of the number of differential metabolites to the total number of metabolites in the pathway. The *y*-axis lists the enriched pathway names. The color gradient of the symbols indicates the enrichment significance, represented by *p*-value (from blue to red, corresponding to decreasing *p*-value and increasing significance). The shape of the symbols indicates the expression direction of differential metabolites: triangles pointing downwards represent pathways enriched with downregulated metabolites, while triangles pointing upwards represent pathways enriched with upregulated metabolites. The size of the symbols indicates the number of differential metabolites (count) in each pathway.

KEGG pathway enrichment analysis demonstrated distinct metabolic reprogramming patterns across dietary crude protein (CP) levels. Between Group I (11.07% CP) and Group II (13.07% CP), significantly enriched pathways (FDR < 0.05) included primary bile acid biosynthesis (ko00120), cholesterol metabolism (ko01100), taurine and hypotaurine metabolism (ko00430), bile secretion (ko04976), and amino sugar and nucleotide sugar metabolism (ko00520). In contrast, the comparison of Group I and Group III (15.11% CP) showed broader and more profound enrichment—spanning steroid biosynthesis (ko00100), oxidative phosphorylation (ko00190), AMPK signaling (ko04152), FoxO signaling (ko04068), steroid hormone biosynthesis (ko00140), and bile secretion (ko04976)—with all pathways achieving FDR < 0.01. For Group II vs. Group III, enrichment was restricted to steroid biosynthesis (ko00100), steroid hormone biosynthesis (ko00140), and alpha-linolenic acid metabolism (ko00632) (FDR < 0.05; Figure 5). Collectively, these results demonstrate a progressive intensification of hepatic metabolic remodeling with increasing dietary CP, particularly in sterol homeostasis, mitochondrial energy transduction, and stress-responsive signaling networks.

### 3.7. Combined Transcriptome–Metabolome Pathway Analysis

#### 3.7.1. Cross-Omics KEGG Pathway Co-Enrichment

Combined pathway mapping demonstrated concordant transcriptional and metabolic perturbations across dietary crude protein (CP) treatments. Between Group I (11.07% CP) and Group II (13.07% CP), significantly co-enriched pathways (FDR < 0.05) included primary bile acid biosynthesis (ko00120), cholesterol metabolism (ko01100), bile secretion (ko04976), taurine and hypotaurine metabolism (ko00430), and PPAR signaling (ko03320). In the Group I vs. Group III (15.11% CP) comparison, co-enrichment extended to biosynthesis of amino acids (ko01230), steroid biosynthesis (ko00100), AMPK signaling (ko04152), oxidative phosphorylation (ko00190), arginine and proline metabolism (ko00330), and bile secretion (ko04976)—with all achieving FDR < 0.01. For Group II vs. Group III, co-enriched pathways were steroid biosynthesis (ko00100), cysteine and methionine metabolism (ko00270), AMPK signaling (ko04152), steroid hormone biosynthesis (ko00140), and tyrosine metabolism (ko00350) (FDR < 0.05). Notably, bile secretion and steroid biosynthesis emerged as recurrent hubs of cross-omics dysregulation across all three pairwise comparisons, suggesting their central role in hepatic adaptation to graded dietary CP.

#### 3.7.2. Spearman Correlation Analysis Between Hepatic Molecular Features and Phenotypic Performance

**Figure 6:**
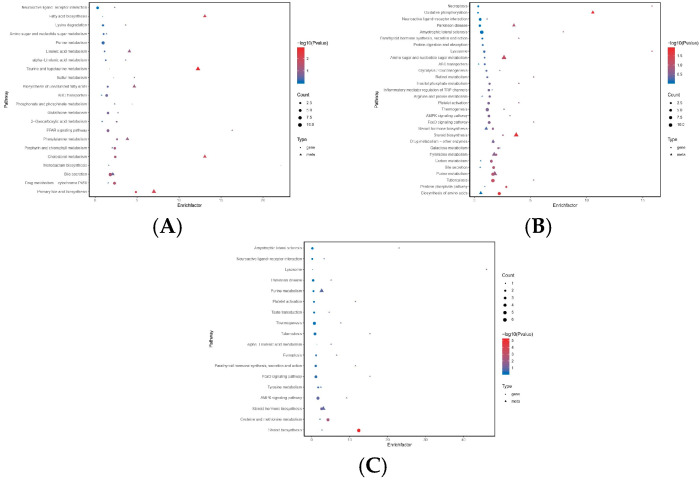
Common Enriched Pathway Map. (**A**) KEGG pathway enrichment bubble plot of differentially expressed genes and metabolites between Group I and Group II. (**B**) KEGG pathway enrichment bubble plot of differentially expressed genes and metabolites between Group I and Group III. (**C**) KEGG pathway enrichment bubble plot of differentially expressed genes and metabolites between Group II and Group III. The vertical axis shows the names of KEGG pathways. The red-to-blue gradient indicates the significance level of enrichment, ranging from high to low, which is quantified by the *p* value. Bubble shapes correspond to different omics layers: circles represent the transcriptome, and triangles represent the metabolome. Bubble size denotes the number of differential genes or metabolites involved in each pathway; a larger bubble indicates a greater number of differential molecules.

**Figure 7:**
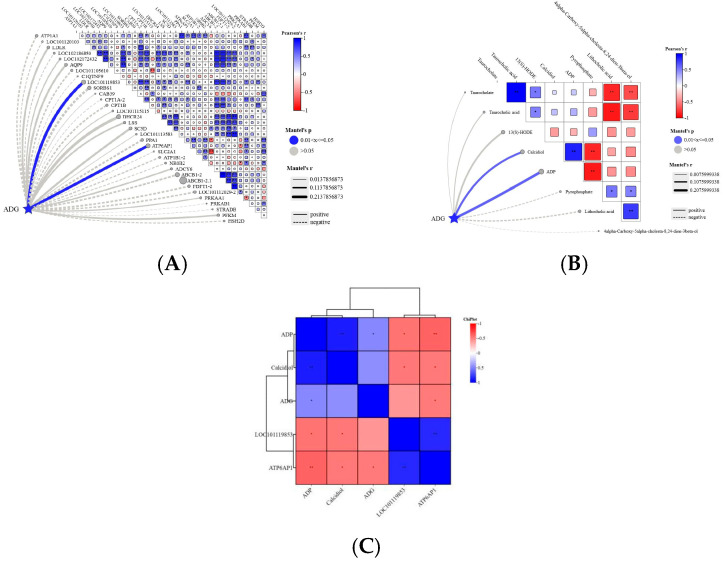
Correlation analysis between average daily gain (ADG) and key genes and metabolites. (**A**) Correlation network diagrams showing the associations between ADG and key genes. (**B**) Correlation network diagrams showing the associations between ADG and key metabolites. The heatmap in the upper right shows pairwise correlations between genes, with color intensity indicating the correlation coefficient (r; blue for positive correlations, red for negative correlations). The network diagram on the left illustrates the Mantel correlation between ADG and each gene, where the line thickness corresponds to the Mantel’s r value, and line type (solid/dashed) indicates positive or negative correlation direction. Blue lines represent correlations with statistical significance (*p* < 0.05), while gray lines indicate non-significant correlations. (**C**) Correlation heatmap of key genes, metabolites, and ADG.The heatmap shows pairwise Pearson correlations between average daily gain (ADG), key genes (LOC101119853, ATP6AP1), and metabolites (ADP, Calcididol). The color gradient indicates the correlation coefficient (r), where blue represents positive correlations and red represents negative correlations. The asterisks in each cell denote the significance level of the correlation. Hierarchical clustering dendrograms are shown on the top and left to illustrate the similarity in correlation patterns. Asterisks denote the significance of correlations: * *p* < 0.05, ** *p* < 0.01.

To explore the molecular relationships linking dietary crude protein (CP) intake to growth performance in Hu sheep, we conducted integrative correlation analysis between differentially expressed genes (DEGs) and metabolites (DEMs) from co-enriched KEGG pathways—and average daily weight gain (ADG). combined pathway mapping across all three pairwise comparisons characterized five recurrently dysregulated pathways: bile secretion (ko04976), AMPK signaling (ko04152), steroid biosynthesis (ko00100), PPAR signaling (ko03320), and oxidative phosphorylation (ko00190) (FDR < 0.05; Figure 6). From these pathways, core DEGs and DEMs were prioritized based on fold-change magnitude, statistical significance, and biological plausibility. Spearman rank correlation analysis demonstrated that two transcripts—ATP6AP1 (r = −0.82, FDR = 0.003) and LOC101119853 (r = −0.79, FDR = 0.006)—exhibited significant negative correlations with ADG; conversely, The metabolites of calcidiol (r = 0.85, FDR = 0.001) and ADP (r = 0.77, FDR = 0.009) were significantly positively correlated with ADG. Critically, ATP6AP1 and LOC101119853 were also significantly inversely correlated with both calcidiol (r = −0.74 and −0.71, respectively) and ADP (r = −0.69 and −0.66), suggesting coordinated transcriptional suppression of key metabolic effectors. These correlation analyses revealed potential molecular associations: at higher dietary crude protein levels, the expression of ATP6AP1 and LOC101119853 was negatively correlated with ADG, and also negatively correlated with the levels of calcidiol and ADP (Figure 7). This pattern suggests a hypothetical molecular network, in which ATP6AP1 and LOC101119853 may have functional connections with the metabolism of calcidiol and ADP through unknown mechanisms, thereby jointly influencing the growth performance of the animals. It should be emphasized that all conclusions are based on correlation analyses and do not represent direct regulation or causal relationships. The conclusions drawn from the calcidiol determined through presumptive identification should be interpreted with caution, and further verification by targeted metabolomics is required.

## 4. Discussion

Dietary crude protein (CP) is a fundamental nutritional determinant affecting growth performance, nitrogen utilization efficiency, and systemic metabolic homeostasis in fattening ruminants [29]. As the central hub for amino acid catabolism, gluconeogenesis, lipid synthesis, and detoxification, the liver serves as the primary molecular interface through which dietary CP modulates phenotypic outcomes in Hu sheep [30,31,32]. A critical limitation of prior studies on Hu sheep protein nutrition lies in the failure to standardize metabolizable energy (ME) concentration—introducing confounding effects from energy–protein interactions [33,34]. Furthermore, the arid, high-UV environment of southern Xinjiang imposes elevated basal metabolic demands and oxidative stress burdens on local Hu sheep, resulting in distinct CP requirements relative to inland populations [35]. Consequently, the molecular basis for appropriate CP intake in this ecotype remains poorly defined—hindering the development of precision-formulated total mixed rations (TMR) for regional production systems. To address this gap, the present study rigorously controlled dietary ME at 9.4 MJ/kg DM and implemented three graded CP levels (11.07%, 13.07%, and 15.11% CP), integrating hepatic transcriptomic and metabolomic profiling to delineate gradual regulatory patterns. This represents the first multi-omics characterization of CP-responsive adaptation in southern Xinjiang Hu sheep, providing both molecular insight and actionable guidance for regional feed formulation.

Consistent with phenotypic data, increasing dietary CP from 11.07% to 15.11% elicited a significant, linear improvement in average daily gain (ADG) and cumulative weight gain (*p* < 0.01), with the 15.11% CP group exhibiting 46.4% higher ADG than the 11.07% CP group. This contrasts with reports from inland regions [36,37], where maximal growth often plateaus at lower CP levels—likely attributable to the heightened demand for antioxidant amino acids (e.g., cysteine, methionine, glycine) and energy substrates required to sustain hepatic redox balance under environmental stress [38,39]. The 15.11% CP level aligns with NRC (2007) recommendations for growing meat sheep and operates within the physiological capacity of hepatic nitrogen metabolism, thereby ensuring adequate amino acid availability for extrahepatic protein synthesis—particularly in skeletal muscle—while optimizing intrahepatic nutrient partitioning toward anabolic processes.

Transcriptomic analysis demonstrated that CP gradients induced systematic, gradual remodeling of the hepatic gene expression landscape. Pathway enrichment patterns were strongly concordant with growth phenotypes: In the 11.07% and 13.07% CP groups, differentially expressed genes (DEGs) were significantly enriched in amino acid biosynthesis (ko01230) and NF-κB signaling (ko04668). Up-regulation of amino acid biosynthesis reflects a compensatory hepatic response to dietary nitrogen insufficiency—diverting ATP and carbon skeletons away from peripheral anabolism toward de novo synthesis [40,41]. Concurrent activation of NF-κB signaling indicates low-grade hepatic inflammation, likely driven by endoplasmic reticulum (ER) stress and mitochondrial ROS accumulation under nutrient scarcity [42,43], establishing a self-reinforcing cycle of “nutrient deficiency→inflammatory activation→growth suppression.” In contrast, the 15.11% CP group exhibited robust enrichment in PI3K-Akt signaling (ko04151), AMPK signaling (ko04152), and glutathione metabolism (ko00480)—forming an combined regulatory axis that coordinates nutrient sensing, cellular energy status, and redox homeostasis to maximize protein accretion efficiency while mitigating oxidative damage [44,45,46].

Metabolomic findings corroborated and extended these transcriptional patterns. Under suboptimal CP supply (11.07% and 13.07%), differentially expressed metabolites (DEMs) were enriched in cholesterol metabolism (ko01100) and primary bile acid biosynthesis (ko00120)—pathways associated with energy-intensive, homeostatic maintenance rather than growth-directed anabolism [47,48]. These shifts coincided with elevated markers of oxidative stress (e.g., decreased reduced glutathione, increased oxidized glutathione). Conversely, the 15.11% CP group displayed pronounced enrichment in steroid biosynthesis (ko00100), oxidative phosphorylation (ko00190), and AMPK signaling (ko04152), indicating a strategic metabolic shift toward active anabolism: enhanced mitochondrial ATP output contributes to protein synthesis fidelity, while steroid hormone precursors (e.g., cholesterol-derived pregnenolone) may potentiate growth hormone–insulin-like growth factor 1 (GH–IGF1) axis activity [49,50]. Notably, lipid metabolism pathways (e.g., fatty acid oxidation, glycerophospholipid metabolism) were enriched in both the 13.07% and 15.11% CP groups—suggesting that moderate-to-high CP intake promotes hepatic lipid flux and membrane integrity, contributing to improved immune competence [51,52].

Exploratory combined transcriptome–metabolome analysis constitutes the core of this work. As CP increased, co-enriched pathways progressively converged upon a tightly coordinated network centered on PI3K-Akt and AMPK signaling—affecting substrate uptake, mitochondrial bioenergetics, redox buffering, and endocrine cross-talk. This multi-layered homeostatic architecture underpins the superior growth performance observed at 15.11% CP. The Spearman correlation analysis further revealed that two transcripts—ATP6AP1 (r = −0.82) and LOC101119853 (r = −0.79)—had a significant negative correlation with ADG. At the same time, they also showed a negative correlation with the levels of the presumed metabolite of calcidiol and ADP. These findings of correlations have led to a testable hypothesis: Under a nutritional condition with abundant crude protein, the relative down-regulation of the expression of ATP6AP1 and LOC101119853 in the liver may be related to maintaining higher levels of calcidiol and ADP, thereby facilitating protein synthesis in skeletal muscle and overall growth efficiency. However, this is merely a biological hypothesis based on correlation analysis, and its specific molecular mechanism awaits validation through functional experiments [53].

Collectively, our data define three functional states across the CP gradient: (i) the 11.07% CP group exhibits minimal adaptive capacity, engaging only basal energy conservation; (ii) the 13.07% CP group occupies a transitional, metabolically compensated state—sustaining viability but not optimized growth; and (iii) the 15.11% CP group achieves a physiologically efficient, homeostatically stable state characterized by synergistic transcriptional–metabolic coordination. Our findings suggest that 15.11% dietary CP contributes to favorable growth performance in Hu sheep reared under southern Xinjiang conditions.

Several limitations should be noted. First, only 4-month-old male lambs were used, limiting generalizability to other ages or sexes. Second, functional validation (e.g., gene knockdown or pathway modulation) was not performed. Third, we analyzed only liver tissue; integrating rumen microbiota, skeletal muscle, or adipose tissue would improve systemic understanding. Fourth, transcriptomic and metabolomic analyses used individual animals as the unit (not pen), potentially underestimating pen-level variation. Fifth, metabolite identifications are putative (MSI Level 2); key metabolites require standard-based quantification. Finally, all findings are correlative and exploratory, not causal. Future studies should include multi-tissue omics, functional assays, and mixed-effect models with pen as a random factor.

## 5. Conclusions

Under standardized dietary metabolizable energy (9.4 MJ/kg DM), a crude protein (CP) concentration of 15.11% contributes to favorable growth performance in 4-month-old confined male Hu sheep reared in southern Xinjiang. This CP level is associated with coordinated transcriptional and metabolic changes in the liver—specifically related to AMPK signaling (ko04152), steroid biosynthesis (ko00100), and oxidative phosphorylation (ko00190)—thereby establishing a robust, multi-layered regulatory network that maintains hepatic metabolic homeostasis across substrate utilization, energy transduction, redox balance, and endocrine signaling. As a result, it achieves dual physiological benefits: enhanced skeletal muscle protein synthesis efficiency and improved systemic resilience to environmental stressors prevalent in arid regions. These findings provide a preliminarily supported, omics-informed framework for precision-formulating total mixed rations (TMRs) for Hu sheep in southern Xinjiang—and offer transferable principles for optimizing protein nutrition in ruminants subjected to climatic stress in arid and semi-arid agroecosystems.

## Figures and Tables

**Table 1:** Ingredient and nutrient composition of the experimental diets (air-dry basis) %.

Items	Trial Group I	Trial Group II	Trial Group III
Ingredients			
Corn stover	23.00	22.00	21.00
Corn	51.70	42.30	40.50
Corn germ meal	4.50	8.40	5.50
Cottonseed meal	3.00	6.50	12.00
DDGS	4.50	7.00	9.00
Corn bran with spray liquor	6.50	7.00	4.10
Gelatinized protein type 200 ^1^	1.00	1.00	1.20
NH_4_Cl	0.40	0.40	0.50
CaCO_3_	1.50	1.50	1.60
NaCl	0.80	0.80	0.80
NaHCO_3_	1.20	1.20	1.20
CaHPO_4_	0.90	0.90	1.20
Vitamin B12	1.00	1.00	1.40
Total	100.00	100.00	100.00
Nutrient levels ^2^			
ME/(MJ/kg)	9.42	9.40	9.42
DM	87.00	87.00	88.00
CP	11.07	13.07	15.11
Ca	0.82	0.82	0.91
P	0.41	0.46	0.53
NDF	25.36	26.44	25.24
ADF	11.80	12.21	12.18

^1^ Type 200 gelatinized protein denotes soybean meal subjected to high-temperature extrusion and steam conditioning, a processing method designed to enhance protein digestibility and reduce anti-nutritional factors. ^2^ ME (metabolizable energy) values were calculated using the NRC (2007) [16] prediction equations for ruminants; all other nutrient concentrations reported in Table 1 were determined experimentally via standardized analytical procedures.

## Data Availability

The data presented in this study are available on request from the corresponding author. The data are not publicly available due to these data may still be used by the research group in the later stage, so they have not been uploaded to the public database.

## Supplementary Materials

The following supporting information can be downloaded at: https://www.mdpi.com/article/10.3390/metabo16060375/s1, Supplementary Figure S1: OPLS-DA Scores Plots and Corresponding Permutation Test Plots.
