# Supplementary material for: Transcriptome and Metabolome Analyses Reveal the Molecular Relationship Between Dietary Crude Protein Level and Liver Metabolism in Fattening Hu Sheep
Source: Metabolites. 2026 May 29;16(6):375. doi: 10.3390/metabo16060375 (PMC13302976; doi:10.3390/metabo16060375)
Supplement: Supplementary file 1 [file metabolites-16-00375-s001.zip › metabolites-4283592-supplementary.pdf]

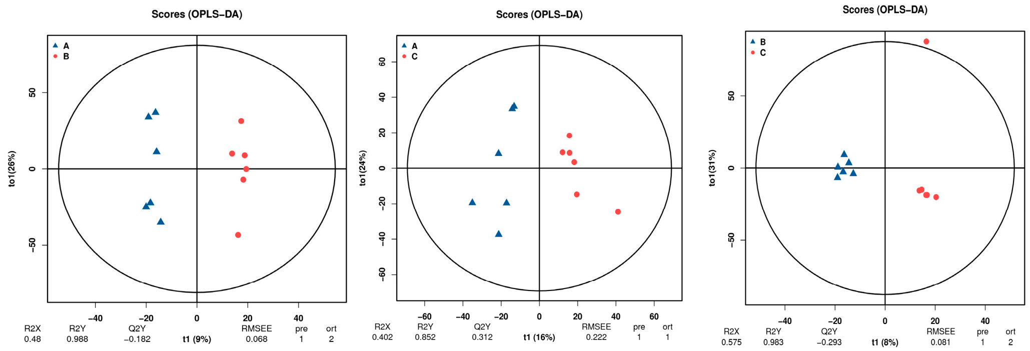


**(A)** **(B)** **(C)**

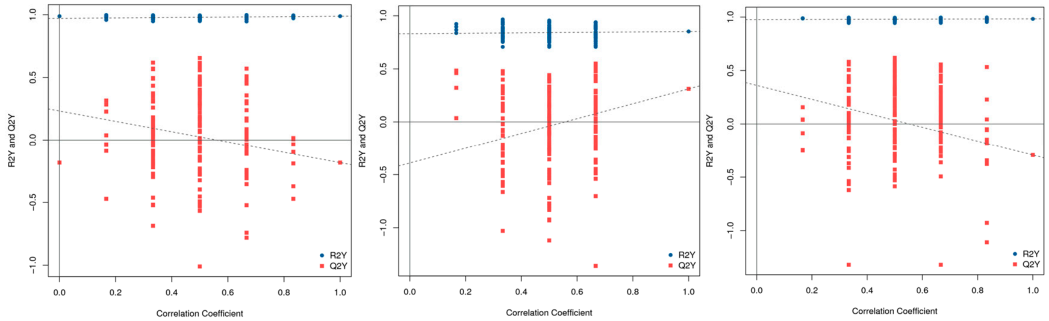


**(a)** **(b)** **(c)**

**Figure S1. (A)** OPLS-DA score plots between Group I (blue triangles) and Group II (red dots). The t1 (9%) and to1 (26%) axes represent the first predictive component and the first orthogonal component, respectively. The ellipse represents the 95% confidence interval (Hotelling's $T^2$). The model parameters are shown below the plot: $R^2X$ = 0.48 (explaining 48% of the X variance), $R^2Y$ = 0.988 (explaining 98.8% of the Y variance), and $Q^2Y$ = -0.182. **(B)** OPLS-DA score plots between Group I (blue triangles) and Group III (red dots)**.** The t1 (16%) and to1 (24%) axes represent the first predictive component and the first orthogonal component, respectively. The ellipse represents the 95% confidence interval (Hotelling's $T^2$). The model parameters are shown below the plot: R2X = 0.402 (explaining 40.2% of the X variance), R2Y = 0.852 (explaining 85.2% of the Y variance), and Q2Y = 0.312. **(C)** OPLS-DA score plots between Group II (blue triangles) and Group III (red dots). The t1 (8%) and to1 (31%) axes represent the first predictive component and the first orthogonal component, respectively. The ellipse represents the 95% confidence interval (Hotelling's $T^2$). The model parameters are shown below the plot: R2X = 0.575 (explaining 57.5% of the X variance), R2Y = 0.983 (explaining 98.3% of the Y variance), and Q2Y = -0.293. **(a)** Permutation test plot of the OPLS-DA model between Group I and Group II. **(b)** Permutation test plot of the OPLS-DA model Group I and Group III. **(c)** Permutation test plot of the OPLS-DA model Group II and Group III. The blue dots represent the $R^2$ values of permuted models, while the red squares represent the $Q^2$ values. The horizontal axis shows the correlation coefficient between permuted and original data, and the vertical axis shows the $R^2$ and $Q^2$ values. The $Q^2$ regression line intersects the y-axis at a negative value, indicating that the original model is not overfitted and has good predictive ability.
